# Predicting Geographic Variation in Cutaneous Leishmaniasis, Colombia

**DOI:** 10.3201/eid1004.030241

**Published:** 2004-04

**Authors:** Raymond J. King, Diarmid H. Campbell-Lendrum, Clive R. Davies

**Affiliations:** * Centers for Disease Control and Prevention, Atlanta, Georgia, USA; † London School of Hygiene and Tropical Medicine, London, United Kingdom

**Keywords:** Colombia, cutaneous leishmaniasis, remote sensing, GIS, predictive modeling, ecological zonation, logistic regression, jackknife

## Abstract

Approximately 6,000 cases of cutaneous leishmaniasis are reported annually in Colombia, a greater than twofold increase since the 1980s. Such reports certainly underestimate true incidence, and their geographic distribution is likely biased by local health service effectiveness. We investigated how well freely available environmental data explain the distribution of cases among 1,079 municipalities. For each municipality, a unique predictive logistic regression model was derived from the association among remaining municipalities between elevation, land cover (preclassified maps derived from satellite images), or both, and the odds of at least one case being reported. Land cover had greater predictive power than elevation; using both datasets improved accuracy. Fitting separate models to different ecologic zones, reflecting transmission cycle diversity, enhanced the accuracy of predictions. We derived measures that can be directly related to disease control decisions and show how results can vary, depending on the threshold selected for predicting a disease-positive municipality. The results identify areas where disease is most likely to be underreported.

## Abstract 2

Predicting Geographic Variation in ACL, Colombia

Transmission of the zoonotic disease American cutaneous leishmaniasis (ACL) is increasing in Latin America (*1*). ACL was originally characterized as an occupational disease of workers, primarily men, exposed to the natural transmission cycle in forests (*2*). Changes in these environments have led to the proliferation of various species of the sand fly vector (*Lutzomyia* spp.), their associated parasites, and reservoirs around rural settlements (*3*,*4*). In some regions, such modifications have facilitated the invasion of vectors that transmit *Leishmania* spp. associated with particularly severe disease (*5*). Peridomestic and domestic transmission have now been recorded in at least nine countries in the Americas (*2*,*6*) and are responsible for an increasing proportion of total cases (*7*,*8*). In the areas subject to most anthropogenic change, ACL now affects all age groups and both sexes almost equally (*9*,*10*).

The impact of ACL may be reduced by the rapid provision of antimonial drugs for treatment (*11*). However, the increasing incidence and domesticity of ACL also increase the feasibility of interventions to interrupt transmission around houses. To date, few control programs have been effective (*12*–*14*). Although interventions such as residual spraying of houses can reduce transmission (14,15), they are rarely applied in a focused, evidence-based manner (*9*). The wide geographic variation in the ecology and behavior of vectors, pathogens, reservoirs, and persons is likely to cause corresponding variation in cost-effectiveness of control measures (*16*). The ecologic and topographic risk factors for ACL in particular geographic regions must be clarified in order for appropriate control methods to be devised and carried out (*17*).

Remote sensing data are increasingly being used to measure environmental and topographic variables on the ground, and geographic information systems (GIS) are being used to model these data both spatially and temporally. The particular advantages of remote sensing include 1) numerous sensors with a wide range of spectral, spatial, and temporal resolutions (*18*,*19*), and 2) global coverage at low or no cost. These properties potentially allow GIS functions to be used to investigate environmental relationships and generate predictive maps throughout wide areas and thus focus control measures (*20*,*21*). These approaches have been used to predict distributions for a wide range of vectors and vector-borne diseases, including sand flies and leishmaniasis (*22*–*24*).

Despite their apparent utility, these techniques are still not widely used by control programs in part because health personnel often consider satellite data difficult to interpret (previous studies have usually used reflectance measurements from satellite sensors or with land-cover classifications “custom-built” by the investigators). The predictive maps that are generated are also often poorly validated and only indirectly related to control decisions. In addition, most previous analyses have been restricted to a relatively limited number of environments, vectors, or parasite species.

We used remote sensing and GIS technologies to investigate the extent to which freely available “off-the-shelf” (i.e., preclassified) land cover and elevation datasets can predict variation in risk for ACL transmission in Colombia, a country characterized by a highly diverse ecology, topography, and climate. These conditions have led to multiple *Leishmania* parasites (6 reported species), mammalian reservoirs (12 reported species), and *Lutzomyia* vectors (12 reported species) (*15*), creating a complex distribution pattern of ACL transmission (*25*).

We used a jackknife method previously used in ecologic studies to test the following factors: 1) the ability of statistical models based on elevation and preclassified land-cover data to predict the probability of ACL transmission in each municipality in Colombia, 2) whether predictive accuracy could be improved by allowing different environmental-disease relationships in different ecologic zones, and 3) the extent to which these predictions could also explain variation in the intensity of transmission (the reported incidence of cases) between disease-endemic municipalities. Finally, we generated various measures of model accuracy and compared their usefulness in terms of informing disease control decisions.

## Methods

### Incidence Data

Data on annual reports of ACL were obtained from the Colombian Ministry of Health. Municipality-level case reports for 1,079 municipalities in 1994 were linked to a georeferenced digitized map of municipality boundaries from the Colombian geographic institute (Instituto Agustin Codazzi) and population information from the 1993 census from the national census organization (DANE), using ESRI ArcView GIS software. This allowed municipalities with at least one reported case to be identified and incidence rates among the rural population to be calculated.

### Explanatory Data: Elevation and Land Cover

The Andean region encompasses wide variations in elevation, which is the principal determinant of variation in temperature, and strongly influences precipitation. Georeferenced elevation data from a 1-km digital elevation model of South America was downloaded from the U. S. Geological Service Earth Resources Observation System (EROS) (*26*). Land-cover data were obtained from the 1-km x 1-km resolution South America Seasonal Land Cover database, accessed from the same source. The data were derived from 1-km resolution, 10-day composites of NOAA-AVHRR satellite images acquired from April 1992 through March 1993, which were classified into land cover types according to their spectral characteristics throughout the year. The resulting land cover map was validated by comparing sample point pixels with cover type identified from Landsat or SPOT images, giving an overall accuracy of 66.9% (*27*).

Of the 167 land-cover classes recorded in South America, 105 were represented in Colombia. However, many of these have similar or identical biologic and ecologic descriptions (Table 1), and the use of a large number of essentially replicated classes makes interpretation difficult and increases the odds of detecting an apparently significant association purely by chance. Classes with identical or highly similar descriptions were therefore grouped together. In four instances when classes were similar or identical, except for a constituent crop associated with ACL transmission (coffee), two classes were created—one with coffee and one without coffee (*28*–*30*). This process generated 25 broader classes.

**Table 1:** Land-cover classification used in the analysis

Identification no.	Land-cover class label
1	Fragmented evergreen forest/grassland/savanna
2	Tropical evergreen rainforest
3	Montane evergreen rainforest
4	Submontane evergreen rainforest
5	Dry deciduous forest
6	Subtropical moist deciduous forest
7	Deciduous woodland
8	Fragmented evergreen forest/cropland
9	Deciduous forest/cropland—includes coffee
10	Fragmented evergreen forest/cropland—includes coffee
11	Cropland—includes coffee/woodland
12	Cropland—includes coffee/savanna/grassland
13	Cropland
14	Cropland/savanna/grassland/pasture
15	Cropland/ woodland
16	Fragmented montane forest/cropland
17	Grassland/savanna/woodland
18	Semiarid deciduous shrub
19	Semiarid thorn shrub/grassland/cropland
20	Flooded grassland
21	Flooded grassland/fragmented forest
22	Flooded evergreen broadleaf forest
23	Andean tundra/shrubland
24	Sparsely vegetated
25	Wooded wetland

Elevation and land-cover data were overlaid on the map of municipality boundaries and incidence rates, using the GIS software TNTmips (MicroImages, Inc., Lincoln, NE). This GIS was used to calculate, for each municipality, the mean elevation and the proportion of total area covered by each land-cover class.

**Figure 1:**
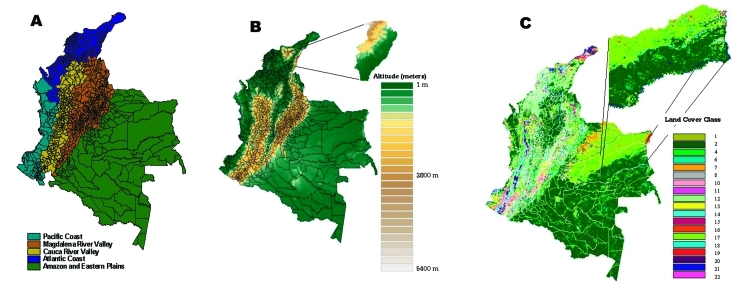
Distribution of a) ecoepidemologic zones, b) elevation, and c) vegetation types in Colombia.

Other mapping studies for tropical diseases (*31*,*32*) have shown that dividing predictive maps into ecologically similar areas can improve their accuracy. The ecologic, topographic, and climatic diversity of Colombia gives rise to 23 distinct vegetative zones (*33*) and a spatially heterogeneous distribution of transmission cycles and intensity of ACL transmission (*15*). For the purposes of this study, we follow Espinal and Montenegro in dividing Colombia into seven ecoregions (*33*). Of these, two small regions were either combined with larger contiguous regions (Catumbo River Basin joined to Magdalena River Valley) or excluded from the analysis (Central Andean Massif). This left five zones for ecoepidemiologic analysis: Pacific, Atlantic, Amazon and Eastern Plains, Cauca River Valley, and Magdalena River Valley. These ecoregions, and the geographic distribution of elevation and vegetation are illustrated in Figure 1.

### Statistical Analysis of Ecologic Associations

Predictions of the probability of transmission were generated by using a jackknife procedure (*34*). In this approach, a single municipality was excluded, and a logistic regression model was fitted to the remaining data. The response variable was defined as either presence or absence of at least one reported case of ACL, and the independent variables were defined as the proportions of the total area of each municipality belonging to each land-cover class, mean elevation, and (to allow nonlinear relationships) the square of the mean elevation. The coefficients from this model were then applied to the values of the predictor variables from the missing municipality to generate a predicted probability of occurrence between 0 and 1. The process was repeated for each municipality. Predicted and observed datasets were therefore independent because the prediction for each municipality was generated by using disease data only from other locations. The statistical significance of the fit was measured by using the chi-square value from a logistic regression of the observed data against the predicted data for all municipalities.

We compared the predictive power of different types of explanatory data using 1) both land cover and elevation information, 2) only elevation information, and 3) only land cover information. To measure the value of dividing the study area into more ecologically homogenous regions, each of the regression procedures was then repeated, but predictions for each municipality were generated by using only data from the same ecologic zone. To assess similarity in ecologic relationships between regions, predictive models from each zone were also used to predict occurrence in all other zones. As the zones are independent, a complete model that included all data from one zone was used to predict the presence or absence in the other zones.

Predictions of presence or absence are often assessed by comparing predictions and observations to measure sensitivity (ability to correctly predict “true” positives), specificity (ability to predict true negatives), positive predictive value (PPV; proportion of predicted positives that are truly positive), negative predictive value (NPV; proportion of predicted negatives that are truly negative) (*35*), and κ statistics (the proportion of observations that we would have expected to be incorrectly predicted on the basis of chance, but which are correctly predicted, i.e., a measure of the additional “skill” of the model over chance).

Because the above procedure predicts a probability of transmission between 0 and 1, a threshold probability must be selected to convert these values into predictions of presence or absence. This selection influences the value of all of the above measures, but this choice is arbitrary unless other factors must be considered, such as differential costs and benefits of identifying positive versus negative locations. We therefore followed a procedure previously used in ecologic and veterinary mapping studies (*36*–*38*), and more recently applied in human disease mapping (*32*), of plotting sensitivity against (1-specificity) for all thresholds between 0 and 1, to generate a receiver-operator curve. The area under the receiver-operator curve (AUC) gives a single comparable measure of overall model performance, reflecting the proportion of occasions on which a randomly selected location with transmission has a predicted probability greater than that for a randomly selected location without transmission. We also calculate sensitivity, specificity, PPV, NPV, and kappa across all thresholds, for both the single model and the combination of the zonal models. Finally, we assessed the ability of the model to predict variation in incidence between disease-endemic municipalities by regressing the log-transformed values for incidence against predictions of probability of transmission, using values from the model that performed best in the above tests.

## Results

### Epidemiologic Data

**Figure 2:**
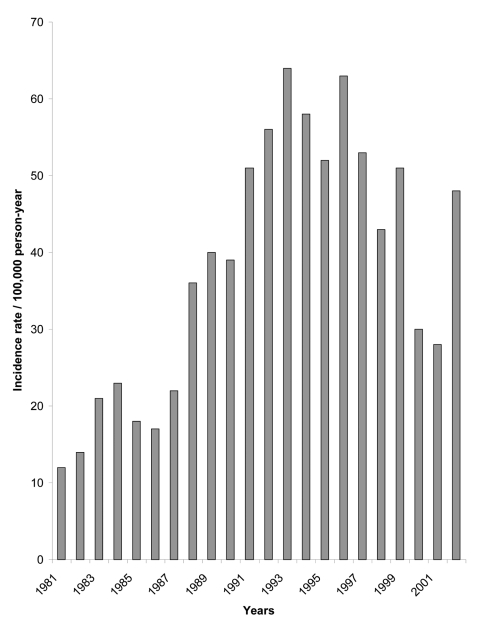
Incidence of American cutaneous leishmaniasis per rural population reported in Colombia by year, 1980–2002 (data from Ministerio de Salud, Colombia).

The reported incidence of ACL (undoubtedly an underestimate of true rates) has more than doubled from the early 1980s to the late 1990s (Figure 2). Table 2 summarizes reported ACL incidence in 1994, and the main parasite and vector species, in each ecoepidemiologic region. Despite very different ecologic characteristics and transmission cycles, each zone has an approximately equal proportion of municipalities reporting ACL transmission.

**Table 2:** Reported incidence of ACL, Colombia, 1994, and major parasite and vector species, by ecoepidemiologic region^a^

Region	Total municipalities	Positive municipalities (% positive)	Median, range of incidence in positive municipalities (/100 000 rural pop.)	Principal vectors	Principal parasite species
Amazon and Eastern Plains	105	42 (40)	62 (7–1,448)	*Leishmania carrerai, L. umbratilis*	*L. amazonensis, L. braziliensis, L. guyanensis, L. mexicana, L. panamensis*
Atlantic	152	50 (33)	57 (2–3,030)	*L. ovallesi*	*L. panamensis*
Cauca River Valley	248	56 (23)	29.5 (3–944)	*L. colombiana, L. trapidoi, L. youngi*	*L. braziliensis, L. panamensis*
Magdalena River Valley	496	136 (27)	64.5 (4–6,662)	*L. gomezi,, L. hartmani, L. longiflocosa, L. ovallesi, L. panamensis, L. spinicrassa, L. torvida*	*L. braziliensis*, *L. panamensis*
Pacific	77	25 (32)	117 (6–1,789)	*L. gomezi, L. trapidoi*	*L. braziliensis, L. mexicana, L. panamensis*

^a^ACL, American cutaneous leishmaniasis; pop., population.

**Figure 3:**
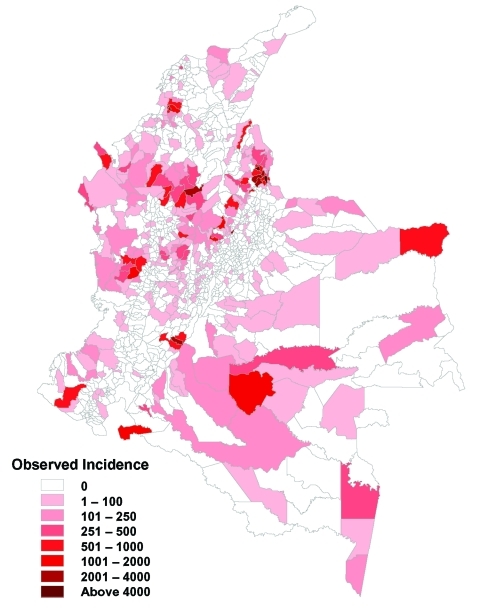
Geographic distribution of American cutaneous leishmaniasis incidence by municipality, 1994

Figure 3 shows the geographic distribution of reported ACL incidence, by municipality. Transmission is absent from the highest elevations along the eastern and western cordilleras of the Andes, presumably because of low temperatures. Elsewhere, transmission is highly heterogeneous, with a small proportion of municipalities reporting a high proportion of the total cases. For example, 50% of the reported cases were from only 20 (1.9%) of all municipalities.

### Environmental Predictions of ACL Transmission

Table 3 summarizes the performance of logistic regression models in predicting the geographic distribution of municipalities with at least one reported case of ACL. Perhaps the best measure of overall model performance is the area under the receiver-operator curve. As a guide, values from 0.5 to 0.7 indicate a poor discriminative capacity, 0.7–0.9 indicate reasonable capacity, and >0.9 indicate a very good capacity. A value of 0.5 is expected by chance (*39*). κ values vary with threshold, so only the maximum κ value is quoted here. κ values below 0.4 can be considered to show poor agreement, 0.4–0.75, good agreement, and above 0.75, excellent agreement (*40*). Values for the other properties also depend on choice of probability threshold, and those shown here correspond to the threshold that gives the highest κ (i.e., where the model has the greatest additional predictive power, above that expected by chance alone).

**Table 3:** Diagnostic statistics of predictive models for presence/absence of ACL transmission^a,b^

Type of model	Predictors used	Accuracy measures					
		AUC	Maximum κ	Sensitivity (%)	Specificity (%)	PPV (%)	NPV (%)
Single model for whole country	Elevation	0.66	0.23	59.9	65.8	41.3	80.3
	Land cover	0.70	0.28	53.4	75.7	46.9	80.2
	All	0.72	0.34	55.3	79.3	51.8	81.6
Combination of zonal models	Elevation	0.70	0.28	53.7	75.2	46.5	80.2
	Land cover	0.82	0.46	67.0	80.6	58.1	85.9
	All	0.84	0.54	62.8	89.1	69.8	85.6

^a^ACL, American cutaneous leishmaniasis; AUC, area under receiver-operator curve; PPV, positive predictive value; NPV, negative predictive value. Sensitivity, specificity, PPV, and NPV are calculated at the probability threshold that gives the highest value of kappa. ^b^For all comparisons of observations against predictions, χ^2^ > 79.2, df =1, p < 0.0001. κ values are given by (proportion correct – Proportion expected)/(1- proportion expected), where proportion correct = (a + d)/n, and proportion expected = (a + b) x (a + c) + (c + d) x (b + d)/n^2^. a = true positive predictions, b = false positive, c = false negative, d = true negative, n = total.

Each modeling approach gives predictions that are significantly better than chance. However, predictions based on zonal division of the country are markedly more accurate than those from a single analysis, demonstrating the advantage of allowing the model to describe different relationships between environment and disease in different ecologic regions. Within either single or zonal modeling approaches, land-cover information from the preclassified satellite images gives greater predictive power than elevation information alone. The most accurate predictions are given by combining both elevation and land cover information.

**Figure 4:**
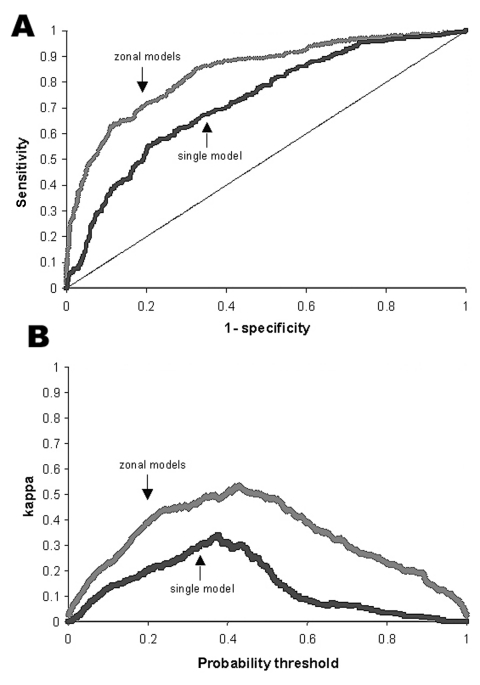
Performance of whole country model versus combination of zonal models. A. Receiver operator curve. Black line, single model for all Colombia (area under the curve [AUC] = 72.4%); gray line, combination of zonal models (AUC = 84.4%). Diagonal line indicates success expected on the basis of chance (AUC = 50%). B. κ value, representing skill at discriminating positive and negative municipalities, above the level expected on the basis of chance. Black line, single model for all Colombia; gray line, combination of zonal models. The probability threshold is the value on the continuous scale of predicted probability of transmission that is used as the cut-off for conversion into a categorical prediction of presence versus absence.

Figure 4 compares the accuracy of predictions from the single model and the combination of the zonal models. Figure 4(A) shows the receiver-operator curve. The improved overall performance of the zonal model is indicated by the curve more closely approaching the top left corner, which represents both maximum sensitivity and specificity. Figure 4(B) shows how the κ statistic varies with choice of threshold, and indicates that both models have greater skill at intermediate probabilities, but that the zonal model has greater skill over a wider range of probability thresholds.

**Figure 5:**
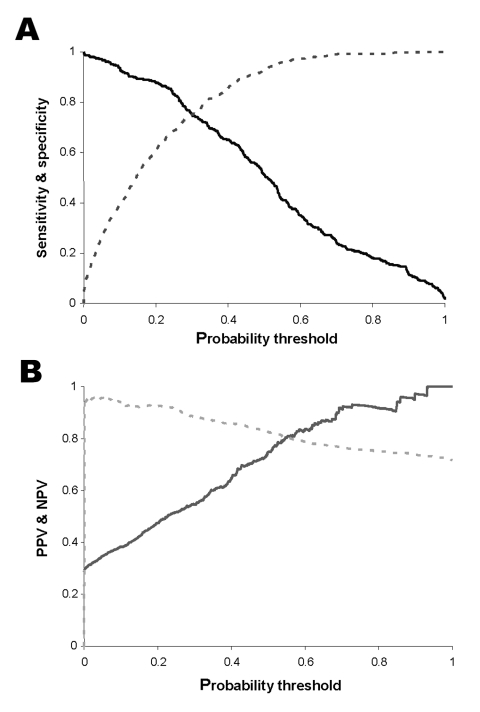
A. Sensitivity (solid line) and specificity (broken line) across the range of threshold probabilities for predicting an endemic municipality. B. Positive predictive value (solid line) and negative predictive value (broken line) across the range of threshold probabilities for predicting a positive municipality. The probability threshold is the value on the continuous scale of predicted probability of transmission that is used as the cut-off for conversion into a categorical prediction of presence versus absence.

Figure 5 uses the predictions from the zonal model to illustrate the effect that the choice of probability threshold has on measures of model accuracy. Figure 5a shows the clear trade-off between sensitivity and specificity: sensitivity is maximized by selecting low threshold values and specificity by selecting high threshold values. Similarly, Figure 5b shows that PPV tends to be higher at greater threshold values, and NPV tends to be higher at lower thresholds, but that the relationship is nonlinear. No single probability threshold optimizes all desirable properties of the predictive model.

Table 4 shows the ability of models generated using data from a single region to predict transmission within the same zone or other zones. The high AUC values on the diagonal confirm that the models are accurate in predicting transmission within the same region. In comparison, they are much less able to predict for ecologically dissimilar zones. Models based on the larger regions (Cauca and Magdalena River Valley) have moderate predictive value for other areas, while those based on smaller regions tend to have poor predictive value and may generate worse predictions than those expected by chance (AUC <50%).

**Table 4:** Accuracy measurement (area under the curve) for models generated using data from one region, assessed within the same region, and in other regions

Assessment region	Model region				
	Amazon and Eastern Plains (%)	Atlantic (%)	Cauca River Valley (%)	Magdalena River Valley (%)	Pacific
Amazon and Eastern Plains	83.4^c^	46.6	52.2	54.7	52.2
Atlantic	45.8	85.2^c^	57.9	56.1	48.2
Cauca River Valley	56.3	51.7	82.2^c^	60.2^b^	50.3
Magdalena River Valley	66.2^c^	56.7	67.7^c^	82.7^c^	52.2
Pacific	59.2	54.5	67.0^a^	70.9^b^	93.6^c^

^a^p < 0.05, ^b^p < 0.01, ^c^p < 0.0001, for fit of predictions against observations.

### Generation of Risk Maps and Comparison with Observed Data

**Figure 6:**
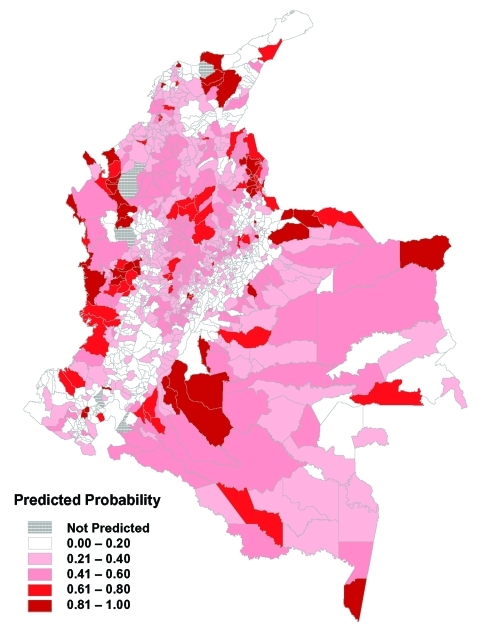
Predicted risk map for probability of transmission, based on the combination of the regional models.

**Figure 7:**
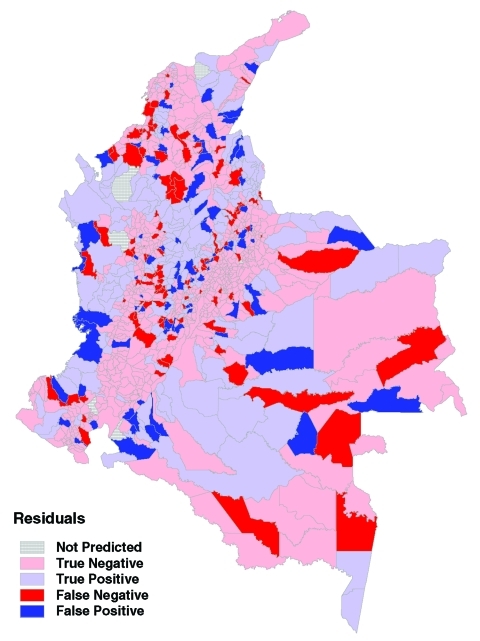
Agreement between predictions and observations. Light blue, correct positive prediction; light red, correct negative; dark blue, false positive; dark red, false negative.

The predictions from the zonal model were used to generate a risk map for probability of transmission occurring in each municipality (Figure 6). The results shows a good match with the observed distribution of transmission (Figure 3). Figure 7 directly compares the two maps. Municipalities were predicted as ACL-endemic or ACL-nonendemic, using the combination of the zonal predictions and applying the probability threshold that gives the maximum value of κ. False-negative predictions (i.e., municipalities where transmission was reported but not predicted by the model) presumably reflect poor ability of the model to describe the effect of local environmental characteristics on transmission risk. False-positive predictions (i.e., transmission was predicted but not reported) may also indicate poor model fit, or alternatively, areas where transmission is occurring but has not been reported.

### Predictions of Variation in Incidence Rates

Regression of log-transformed incidence data in positive municipalities against predicted probability of disease transmission showed a highly significant positive correlation (ln [incidence] = 3.43 + 1.51 x predicted probability, F_1,305_ = 19.04, p < 0.001), indicating that the models also have some value in predicting transmission intensity. However, the regression explained only a small proportion of the variance (6%), and did not describe the true range of variation in incidence, whereas the fitted line gave predictions of log incidence between 3.43 and 4.94 (corresponding to incidence rates of 31 to 140 per 100,000 rural population); observed incidence rates ranged between 2 and 6,662 per 100,000.

## Discussion

During the last decade or so, multiple studies have shown the utility of remote sensing and GIS analysis for identifying the ecologic determinants of the distributions of parasitic diseases, and thereby generating predictive maps. For disease control programs to apply and act on these techniques, however, the resulting predictive maps should 1) give accurate predictions against independent data, 2) be based on predictor data that are inexpensive and easy to interpret, and 3) generate outputs that are directly related to control decisions.

The modeling and validation procedure used here has not previously been used in mapping vector-borne disease. However, previous ecologic applications highlight several advantages over alternative methods, such as using the full dataset for both model development and validation (which overestimates predictive accuracy), or dividing the dataset into independent “training” and validation sets (which involves a subjective decision over division of data, and a reduction in sample size) (*34*). Each prediction generated here is based on the maximum sample of independent data and reflects the process of attempting to predict the next observation in an accurate and unbiased manner.

Application of this technique shows that readily available land cover maps, preclassified from NOAA-AVHRR satellite data, can help accurately predict the presence or absence of ACL transmission at the municipality level across a large, ecologically and geographically diverse country. These maps have a substantial advantage in that they can be easily manipulated and interpreted by persons with basic GIS skills, ecologic knowledge, and computer equipment, without requiring detailed technical knowledge of the properties of satellite sensors or the reflectance properties of different land-cover types.

As in other mapping studies of ecologically diverse areas (*31*,*32*), the accuracy of model predictions is improved when different statistical models are applied in distinct areas. Although the zones used here are defined by using general ecologic characteristics, future models could potentially be further improved by defining zones based specifically on the distribution of the principal sand fly vectors.

The remaining prediction errors are likely due to several factors. These include the spatial resolution of the AVHRR data and accuracy of classification into land cover types, the procedure for grouping into larger classes, and the slight difference between dates of collection of satellite and disease data. The factors also include nonecologic explanatory variables not captured in the model, such as variation in human behavior and housing quality. Alternatively, the errors may reflect limitations in the surveillance system, such as unreliable diagnosis and notification, or cases acquired in municipalities other than where they were diagnosed and reported. The models generated here are relatively poor at predicting the variation in transmission intensity within positive municipalities. The reasons for this are unclear; the characteristics captured in our models may be useful in defining the minimum ecologic conditions necessary for transmission, but other influences such as the demographics, behavior, and herd immunity of human and reservoir populations may exert a stronger influence on incidence rates within these ecologically suitable areas.

The techniques used here have two main practical applications. First, they allow simple hypotheses about the major determinants of disease distributions to be tested. For a country with a large range in altitude, elevation has relatively poor predictive power compared to land cover. Alternative elevation measures (such as minimum elevation, terrain roughness, average slope) might provide more explanatory value. Because land cover is partly determined by elevation, the various land-cover classes described here do effectively integrate elevation data with other ecologic influences, such as latitude, rainfall, and human influences on the environment. Nevertheless, both types of data improve predictive accuracy, and merit collection and application in risk mapping.

The use of preclassified land-cover data facilitates biologic interpretation and suggests what may happen with specific future land use changes (e.g., the replacement of forest with common crops). The analysis could be further developed by updating with more recent land-cover information, by applying species-specific information on the relationship between vectors and vegetation in order to refine the grouping of land-cover classes, and by investigating whether particular land-cover types are associated not only with incidence but also with the level of peridomestic transmission, as indicated by relative infection rates in children versus adults, or by the abundance of sand fly vectors with known domestic behavior. Our approach can also serve as a “first-cut” to identify areas of particular interest (either high transmission risk or with specific ecologic associations) that could be further investigated by using satellite imagery with greater spatial resolution (*19*).

The second practical application of these datasets and analyses is for generating predictive maps that can be used to target resources (e.g., drugs or insecticide spraying activity) between municipalities when notification data are incomplete or unreliable. No risk map is 100% accurate, and a range of statistics are available for assessing model quality. AUC is an appropriate measure for comparing the overall performance of different models. However, comparisons should ideally be made in relation to a specific control decision and with a priori knowledge of the particular characteristics that the control program is attempting to optimize. For example, sensitivity indicates the probability of correctly identifying a disease-endemic municipality. If sensitivity is low, many communities at high risk will not receive the resources they require. In contrast, PPV measures the probability that a community, which we predict to have high risk, is truly at high risk. If PPV is low, a risk map could lead to a waste of resources and unnecessary environmental or health damage as the result of insecticide spraying. Therefore, the final assessment of the utility of a risk map requires a full analysis of the relative economic costs and health benefits of decisions made based on its predictions.

An apparent paradox exists in that predictive mapping attempts to estimate disease risk in areas with poor or missing data, yet all mapping studies ultimately rely on the quality of the underlying data. More and higher quality data should lead to improved hypothesis testing and predictions, yet as the quantity and quality of the reporting data improve, predictions are needed less. Predictive modeling and data collection may therefore be most productive when used as complementary iterative processes, with models highlighting where new data collection is most important, and surveillance data are continually improving the models. In this process, errors are more informative than correct predictions. In our example, prediction errors indicate either sites where transmission occurs but is not predicted (suggesting different ecologic relationships requiring further research) or sites where transmission is predicted but not observed (possibly representing under-reporting, and therefore priority areas when revising surveillance systems). Predictive mapping is not a replacement for ecologic fieldwork and reliable reporting systems but can be a useful tool for directing each of these fundamental activities and extracting maximum value from them.
